# Comprehensive Geriatric Assessment: Application and correlations in a real-life cross-sectional study

**DOI:** 10.3389/fmed.2022.984046

**Published:** 2022-09-13

**Authors:** Francesco Salis, Simona Loddo, Francesca Zanda, Maria Monica Peralta, Luca Serchisu, Antonella Mandas

**Affiliations:** ^1^Department of Medical Sciences and Public Health, University of Cagliari, Cagliari, Italy; ^2^University Hospital “Azienda Ospedaliero-Universitaria” of Cagliari, Cagliari, Italy

**Keywords:** elderly, real-life, Comprehensive Geriatric Assessment (CGA), nutritional status, Mini Nutritional Assessment (MNA)

## Abstract

**Background:**

The assessment process of elderly people considers all aspects of an individual’s life, including physical, mental, and social aspects. Frailty refers to a decline in physiological functions or strengths leading to increased vulnerability to stressors and decreased ability to cope with them. Comprehensive Geriatric Assessment (CGA) is a validated and useful tool in this context to holistically study elderly people. The primary aim of this study was to determine the prevalence of impaired health status in a large geriatric population turning to outpatient service, based on the components of the CGA, and thus to describe its usefulness in real-life clinical practice. The secondary aim of this study was the evaluation of the association between nutritional status, assessed with Mini Nutritional Assessment (MNA)—within the CGA—and cognitive-affective and functional capacities, and multimorbidity.

**Materials and methods:**

This real-life, retrospective cross-sectional study included subjects consecutively evaluated from January 2009 to December 2020 at the Geriatric Outpatient Service, University Hospital of Monserrato, Cagliari, Italy. A sum of 3,260 patients were subjected to CGA.

**Results:**

Only a small proportion of the sample (2.24%) showed an absence of impairment in cognitive-affective, functional, and nutritional domains. Moderate correlations were found between MNA and several other CGA variables, namely, Geriatric Depression Scale (GDS; ϱ = −0.41, *p* < 0.0001), Barthel Index of Independence in Activities of Daily Living (ADL) (ϱ = 0.51, *p* < 0.0001), Instrumental Activities of Daily Living (IADL) (ϱ = 0.43, *p* < 0.0001), and Performance-Oriented Mobility Assessment (ϱ = 0.44, *p* < 0.0001). A multiple regression also highlighted these variables as significant regressors of MNA. Finally, malnutrition showed a significant association with depression (odds ratio [OR]: 4.97), dependence on ADL (OR: 19.8) and IADL (OR: 7.04), and falling risk (OR: 5.16).

**Conclusion:**

This study has figured out the complex situation in which geriatric care finds itself the complexity and severe impairment of elderly people. The possibilities of intervention are often limited, but the literature confirms the benefits of good nutritional status on the general health status. The data that emerged from our study fit into this assumption, highlighting the close association between the nutritional domain and the other CGA domains.

## Introduction

With a higher life expectancy, and general health awareness on the rise, the world is witnessing an increase in geriatric patients (1). Moreover, it is known that Italy, and especially Sardinia, is one of the countries with a larger prevalence of elderly people (2). Primary care providers and geriatricians have tailored their diagnostic and therapeutic practices toward this section of the population. More articles in scientific literature deepen the specifics of assessment markers and how predictive tools can assist in improving these patients’ quality of life (1, 3, 4). In this regard, Comprehensive Geriatric Assessment (CGA) represents a specialistic tool that takes a deep look into several areas, i.e., cognitive, mood, functional status, nutrition, social and interpersonal relationship, and caregivers’ status in order to holistically frame, and thus help, the patient, where possible (3–6). The years have yielded evidence that make CGA essential in clinical practice, as long as it is exploited when there is still room for maneuver; otherwise, it may turn into a useful tool to take note of a critical clinical condition and, where appropriate, the receipt of indemnity, but mournfully not to significantly improve the patient’s health and quality of life (7). At the current state of the art, namely, an advanced cognitive impairment on organic or vascular basis, affecting a high percentage of subjects from 5 to 7% in most countries (8), does not find treatments to reverse the history of the disease (9) though some recent studies encourage research in this direction (10–12). Similarly, a severe dependence on the execution of daily activities can hardly be restored—this condition also affects a considerable proportion of the geriatric population (13). That is, the real-life use of CGA as a diagnostic tool should be preceded by a standardized selection of patients who can benefit from it (7). Nevertheless, evidence has been produced that adequate interventions in single CGA domains can also improve the performances in other domains (14). This applies, in particular, to the nutritional status, an important actor of the health and wellbeing of elderly people, and also considers one of the most important factors involved in the complex etiology of sarcopenia and frailty (14). For its part, due to many factors, such as frailty, multimorbidity, polypharmacotherapy, and inappropriate use of drugs (15), nutritional intake is often compromised. The prevalence of malnutrition is generally < 10% in the elderly people who live in their house and increases up to two-thirds of the elderly admitted to the acute ward or rehabilitation facilities (16). The risk of malnutrition is similar in different settings, i.e., generally ≥ 40% (16). A recent global consensus approach [Global Leadership Initiative on Malnutrition (GLIM)] defines malnutrition as the combination of at least one phenotype criterion, i.e., non-volitional weight loss, low body mass index (BMI), and reduced muscle mass, and one etiologic criterion, i.e., reduced food intake/malabsorption or severe disease with inflammation (17). Furthermore, a close relationship between malnutrition and negative outcomes is well documented in the elderly, e.g., the frequency of infections or pressure ulcers and the length of hospitalization and convalescence following an acute disease (14). In particular, in patients with chronic diseases, malnutrition represents an independent risk factor for increased mortality (18).

Moreover, recent studies suggest that malnutrition is associated with cognitive decline and the degree of impairment in daily functioning in patients with dementia (19). The above epidemiological, clinical, and pathophysiological aspects underline the importance of periodical screening. Given this necessity, new studies are needed to understand the real-life state of the geriatric population and to study and deepen the correlations between the domains of geriatric assessment. This study will try to answer these questions with a cross-sectional approach. It fits into a thriving field of study, contributing to explain the growing burden of multimorbidity and demand for care.

The primary aim of this study was to determine the prevalence of impaired health status in a large geriatric population turning to outpatient service, based on the components of the CGA, and thus to describe its usefulness in real-life clinical practice.

The secondary aim of this study was the evaluation of the association between nutritional status, assessed with MNA—within the CGA—and cognitive-affective and functional capacities, and multimorbidity.

This study fits into a thriving field of study, contributing to explain the growing burden of multimorbidity and demand for care.

## Materials and methods

### Design of the study

This real-life, retrospective cross-sectional study included subjects consecutively evaluated from January 2009 to December 2020 at the Geriatric Outpatient Service, University Hospital of Monserrato, Cagliari, Italy.

Inclusion criteria: It includes those with the age of ≥65 years and has been subjected to CGA from January 2009 to December 2020.

Exclusion criteria: It includes those with the age of <65 years and has not been subjected to CGA.

A total of 3,260 subjects met the inclusion criteria.

### Assessment

The enrolled subjects were evaluated with a validated battery of tools, including

Mini-Mental State Examination (MMSE), which is a screening test to detect severity and changes in time of cognitive impairment. The total score, given by the sum of the exact answers in each item, corrected for age and education, with scores from 30 (absence of cognitive impairment) to 0 (maximum cognitive impairment). A score of <24 is suggestive of cognitive impairment (20, 21).

Geriatric Depression Scale (GDS): It is a screening tool designed to evaluate the presence of depression in elderly subjects with MMSE > 14. This test is made up of 15 Yes/No questions, each of which is translated into a relative score of “0” or “1” as a result of the absence/presence of the investigated depressive symptom. The score ranges from 0 (absence of depression) to 15. A score of >5 is suggestive of depression (22).

Barthel Index of Independence in Activities of Daily Living (ADL): It is used to assess the ability to perform tasks such as taking a bath, using the toilet, walking, maintaining urinary and fecal continence, dressing up, and feeding. The total score ranges from 100 (independence) to 0 (complete dependence) (23). In its modified version, the various grades of dependence are distinguished as follows: 0–24 (complete dependence), 25–49 (severe dependence), 50–74 (moderate dependence), and 75–90 (mild dependence) (24).

Instrumental Activities of Daily Living (IADL): It is used to assess the ability to perform tasks such as using a telephone, doing laundry, and handling finances. The score ranges from 8 (independence) to 0 (complete dependence). A score of <6 is suggesting of dependence (25).

Mini Nutritional Assessment (MNA): It is a tool to assess malnutrition or its risk. The total score is obtained from the sum of the scores assigned to the answers of the 18 questions, which can be divided into four sections (anthropometric, global, dietetic, and subjective). The total score classifies the subject as follows: malnourished (<17), risk of malnutrition (17–23.5), and well-nourished (≥24) (26).

Performance-Oriented Mobility Assessment (POMA): It is a tool for the evaluation of balance and gait in the elderly without cognitive impairment or affected by mild-to-moderate dementia. It identifies subjects at falling risk. The total score (0–28) is obtained by adding the resulting partial scores of two sections, namely, Balance and Gait. The total score classifies the subject in the following way: not able to walk (<2); high fall risk (2–19); moderate fall risk (20–23); and not increased fall risk (≥24) (27).

Cumulative Illness Rating Scale (CIRS): It is a tool to measure the elderly state of health. It evaluates 14 categories of pathologies concerning some organs and systems (e.g., heart vascular system, lungs, liver, and kidneys), hypertension, and psychiatric and behavioral aspects. Every item is evaluated on an ordinal scale with increasing severity levels, from 1 (absence of pathology) to 5 (severe pathology). Using this tool, we can evaluate the total score (CIRS Tot.), the Severity Index (CIRS ISC), which is obtained from the average of the 13 categories scores—excluding psychiatric and behavioral problems—, and the Comorbidity Index Score (CIRS ICC), which corresponds to the number of categories with a score of ≥3 (28).

### Statistical analysis

Quantitative variables were expressed as median and interquartile ranges (IQRs). The Mann–Whitney test for continuous variables was used to study gender differences in CGA variables. Spearman’s rank correlation coefficient (ϱ) and, since this is a retrospective study, odds ratios (ORs) were used to study the relationship between the variables. The Kruskal–Wallis test was used to compare the three groups deriving from MNA scores. The Conover test was performed for *post-hoc* analysis. Multivariate analysis was performed with a stepwise multiple regression (*p*-values > 0.1 were excluded by the model): MNA scores were considered as “dependent variable”; the remaining CGA scores (MMSE, GDS, ADL, IADL, POMA, CIRS Tot., CIRS ICC, and CIRS ISC), age, and gender were considered “independent variables”.

The results are reported that indicate *p*-values in reference to 95% confidence intervals.

The MedCalc software (version 19.5; Ostend, Belgium) was used for the statistical analysis.

## Results

This retrospective cross-sectional study included 3,260 participants, of whom 2,350 (72.1%) were women. The characteristics of the enrolled subjects are shown in Table 1. Comorbidities are summarized in Table 2.

**TABLE 1:** Characteristics of the subjects.

			Gender M	Gender F	Mann–Whitney
Variables	Median (IQR)	Min – Max	Median (IQR)	Median (IQR)	*P*
Age (years)	81 (76–85)	65 – 101	81 (75–85)	81 (76–85)	**0.6054**
MMSE	21.4 (16–25.5)	0 – 30	21.7 (16.2–26)	21.3 (16–25.4)	**0.1084**
GDS	9 (5–12)	0 – 15	7 (4–10)	9 (6–12)	<0.0001
ADL	71 (53–85)	0 – 100	73 (54–88)	70 (53–84)	0.0003
IADL	2 (1–4)	0 – 8	1 (1–3)	2 (1–4)	<0.0001
MNA	20.5 (17.5–23)	0 – 30	21 (18–24)	20 (17.5–23)	0.0001
POMA	14 (9–19)	0 – 28	15 (10–21)	13 (9–18)	<0.0001
CIRS Tot.	32 (29–35)	7 – 45	32 (29–35)	33 (28–34)	0.0014
CIRS ICC	7 (5–8)	0 – 13	7 (5–8)	7 (5–8)	**0.1343**
CIRS ISC	2.23 (2–2.43)	1 – 3.23	2.23 (2–2.46)	2.23 (2–2.43)	0.0007

IQR, Interquartile Range; M, male gender; F, female gender; MMSE, Mini-Mental State Examination; GDS, Geriatric Depression Scale; ADL, Barthel Index of Independence in Activities of Daily Living; IADL, Instrumental Activities of Daily Living; MNA, Mini Nutritional Assessment; POMA, Performance-Oriented Mobility Assessment; CIRS Tot., Cumulative Illness Rating Scale Total score; CIRS ICC, Cumulative Illness Rating Scale Comorbidity Index; CIRS ISC, Cumulative Illness Rating Scale Severity Index.

**TABLE 2:** Comorbidities.

Co-morbilities	Percentage
Geriatric syndromes*	98%
Hypertension	74%
Previous myocardial infarction	15%
Peripheral vascular disease	34%
Chronic cerebrovascular disease	30%
Dementia	17%
Chronic obstructive pulmonary disease	23%
Osteoarthritis	60%
Active neoplasia	10%
Chronic kidney disease	16%
Endocrine disease	22%

*Cognitive impairment, depression, functional dependence, malnutrition.

Table 1 also shows gender differences in performing CGA: women seemed to be more vulnerable than men in GDS, ADL, CIRS Tot., MNA, and POMA (*p* < 0.0001), while no gender-related differences were found in age, MMSE, and CIRS ICC.

**FIGURE 1:**
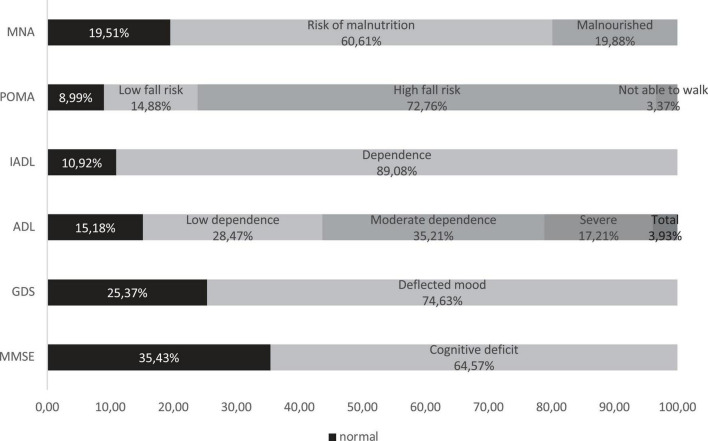
Comprehensive Geriatric Assessment. MMSE, Mini-Mental State Examination; GDS, Geriatric Depression Scale; ADL, Barthel Index of Independence in Activities of Daily Living; IADL, Instrumental Activities of Daily Living; POMA, Performance-Oriented Mobility Assessment; MNA, Mini Nutritional Assessment.

Figure 1 shows the percentage of patients who were found to be compromised on CGA variables: cognitive impairment was suspected in 64.6%, and depression was suspected in 74.6%. Regarding nutritional assessment, it was poor in 80.5% of the subjects (19.9% showed malnourishment, and 60.6% was considered at risk of malnutrition). Total dependence on ADL was found in 3.9% of patients, severe dependence in 17.1%, and moderate dependence in 35.2%; regarding IADLs, 89.1% of patients needed assistance in completing them. Lastly, POMA revealed a high risk of falling in 72.8% of participants (3.4% was not able to walk).

Only 73 (2.24%) subjects have proved good performances in MMSE, GDS, ADL, IADL, and MNA.

A sum of 1,819 (55.8%) people were revealed dependent on ADL (e.g., moderate, severe, and total dependence) and IADL, but only 495 (27.2% of them) could be considered cognitively intact.

The correlation between the variables has been studied using Spearman’s rank correlation coefficient (ϱ), and the results are shown in Table 3. Very strong correlation (0.8 < ϱ < 0.99) was found between CIRS Tot., CIRS ICC, and CIRS ISC; strong correlation (0.6 < ϱ < 0.79) was found between ADL and IADL (ϱ = 0.63, *p* < 0.0001), ADL and POMA (ϱ = 0.71, *p* < 0.0001), and MMSE and IADL (ϱ = 0.53, *p* < 0.0001); moderate correlation (0.4 < ϱ < 0.59) was found between MNA and ADL (ϱ = 0.51, *p* < 0.0001), MNA and IADL (ϱ = 0.43, *p* < 0.0001), MNA and POMA (ϱ = 0.44, *p* < 0.0001), and MNA and GDS (ϱ = −0.41; *p* < 0.0001). The other correlations were weak or very weak (ϱ < 0.4) or not statistically significant.

**TABLE 3:** Spearman’s rank correlation.

		ADL	CIRS Tot.	CIRS ICC	CIRS ISC	Age	GDS	IADL	MMSE	MNA	Gender	POMA
**ADL**	ϱ		−0.379	−0.341	−0.361	−0.205	−0.318	0.629	0.298	0.512	0.064	0.712
	p		<0.0001	<0.0001	<0.0001	<0.0001	<0.0001	<0.0001	<0.0001	<0.0001	0.0003	<0.0001
**CIRS Tot.**	ϱ	−0.379		0.894	0.978	0.013	0.289	−0.198	−0.06	−0.28	0.059	−0.386
	p	<0.0001		<0.0001	<0.0001	**0.4793**	<0.0001	<0.0001	0.0012	<0.0001	0.0014	<0.0001
**CIRS ICC**	ϱ	−0.341	0.894		0.885	0.024	0.266	−0.153	−0.005	−0.247	0.026	−0.33
	p	<0.0001	<0.0001		<0.0001	**0.1745**	<0.0001	<0.0001	**0.7765**	<0.0001	**0.1343**	<0.0001
**CIRS ISC**	ϱ	−0.361	0.978	0.885		0.006	0.247	−0.166	0.007	−0.259	0.059	−0.377
	p	<0.0001	<0.0001	<0.0001		**0.7429**	<0.0001	<0.0001	**0.6768**	<0.0001	0.0007	<0.0001
**Age**	ϱ	−0.205	0.013	0.024	0.006		−0.1	−0.251	−0.18	−0.114	−0.009	−0.192
	p	<0.0001	**0.4793**	**0.1745**	**0.7429**		<0.0001	<0.0001	<0.0001	<0.0001	**0.6054**	<0.0001
**GDS**	ϱ	−0.318	0.289	0.266	0.247	−0.1		−0.219	−0.164	−0.409	−0.185	−0.307
	p	<0.0001	<0.0001	<0.0001	<0.0001	<0.0001		<0.0001	<0.0001	<0.0001	<0.0001	<0.0001
**IADL**	ϱ	0.629	−0.198	−0.153	−0.166	−0.251	−0.219		0.533	0.434	−0.125	0.388
	p	<0.0001	<0.0001	<0.0001	<0.0001	<0.0001	<0.0001		<0.0001	<0.0001	<0.0001	<0.0001
**MMSE**	ϱ	0.298	−0.06	−0.005	0.007	−0.18	−0.164	0.533		0.255	0.028	0.145
	p	<0.0001	0.0012	**0.7765**	**0.6768**	<0.0001	<0.0001	<0.0001		<0.0001	**0.1084**	<0.0001
**MNA**	ϱ	0.512	−0.28	−0.247	−0.259	−0.114	−0.409	0.434	0.255		0.068	0.438
	p	<0.0001	<0.0001	<0.0001	<0.0001	<0.0001	<0.0001	<0.0001	<0.0001		0.0001	<0.0001
**Gender**	ϱ	0.064	0.059	0.026	0.059	−0.009	−0.185	−0.125	0.028	0.068		0.095
	p	0.0003	0.0014	**0.1343**	0.0007	**0.6054**	<0.0001	<0.0001	**0.1084**	0.0001		<0.0001
**POMA**	ϱ	0.712	−0.386	−0.33	−0.377	−0.192	−0.307	0.388	0.145	0.438	0.095	
	p	<0.0001	<0.0001	<0.0001	<0.0001	<0.0001	<0.0001	<0.0001	<0.0001	<0.0001	<0.0001	

*p* > 0.05 highlighted in bold; gender: 0, female. ϱ, Correlation Coefficient; MMSE, Mini-Mental State Examination; GDS, Geriatric Depression Scale; ADL, Barthel Index of Independence in Activities of Daily Living; IADL, Instrumental Activities of Daily Living; MNA, Mini Nutritional Assessment; POMA, Performance-Oriented Mobility Assessment; CIRS Tot., Cumulative Illness Rating Scale Total score; CIRS ICC, Cumulative Illness Rating Scale Comorbidity Index; CIRS ISC, Cumulative Illness Rating Scale Severity Index.

The study population was divided into three groups according to MNA scores, namely, MNA1 (score ≥ 24), MNA2 (24–17), and MNA3 (<17). The Kruskal–Wallis test was conducted to examine the differences in CGA scores according to the nutritional status. Significant differences (*p* < 0.000001) were found among the three groups (Table 4). *Post-hoc* analysis, conducted with the Conover test, showed a decrease in average ranks for MMSE, ADL, IADL, and POMA, and an increase in average ranks for age, GDS, CIRS Tot., CIRS ICC, and CIRS ISC, in MNA3 vs. MNA2 and MNA1, and in MNA2 vs. MNA1 (Table 5).

**TABLE 4:** Kruskal–Wallis test.

Variables	MNA1	MNA2	MNA3	Kruskal–Wallis
	Median (IQR)	Median (IQR)	Median (IQR)	P
**Age (years)**	79.5 (75–84)	81 (76–85)	82 (77–86)	<0.000001
**MMSE**	23.8 (19.3–26.7)	21.5 (16.2–25.45)	18.4 (12.85–23.2)	<0.000001
**GDS**	5 (3–9)	9 (6–12)	11 (9–13)	<0.000001
**ADL**	89 (78.5–96)	69 (53–82)	53 (36–71)	<0.000001
**IADL**	4 (2–6)	2 (1–3)	1 (0–3)	<0.000001
**POMA**	20 (15–24)	13 (9–18)	10 (7–14)	<0.000001
**CIRS Tot.**	30 (26–33)	32 (29–35)	33 (30–36)	<0.000001
**CIRS ICC**	6 (4–7)	7 (5–8)	7 (6–9)	<0.000001
**CIRS ISC**	2.07 (1.85–2.3)	2.23 (2–2.43)	2.31 (2.08–2.53)	<0.000001

IQR, Interquartile Range; MMSE, Mini-Mental State Examination; GDS, Geriatric Depression Scale; ADL, Barthel Index of Independence in Activities of Daily Living; IADL, Instrumental Activities of Daily Living; MNA, Mini Nutritional Assessment; POMA, Performance-Oriented Mobility Assessment; CIRS Tot., Cumulative Illness Rating Scale Total score; CIRS ICC, Cumulative Illness Rating Scale Comorbidity Index; CIRS ISC, Cumulative Illness Rating Scale Severity Index; MNA1, MNA score ≥ 24; MNA2, MNA score 23.5–17; MNA3, MNA score < 17.

**TABLE 5:** Conover test.

Variables		Average rank	Different from
Age	MNA1	1488.11	MNA2, MNA3
	MNA2	1622.1	MNA1, MNA3
	MNA3	1785.78	MNA1, MNA2
MMSE	MNA1	1961.66	MNA2, MNA3
	MNA2	1641.19	MNA1, MNA3
	MNA3	1262.89	MNA1, MNA2
GDS	MNA1	853.17	MNA2, MNA3
	MNA2	1467	MNA1, MNA3
	MNA3	1826.71	MNA1, MNA2
ADL	MNA1	2466.44	MNA2, MNA3
	MNA2	1553.87	MNA1, MNA3
	MNA3	1033.2	MNA1, MNA2
IADL	MNA1	2314.62	MNA2, MNA3
	MNA2	1577.4	MNA1, MNA3
	MNA3	1110.59	MNA1, MNA2
POMA	MNA1	2328.75	MNA2, MNA3
	MNA2	1566.76	MNA1, MNA3
	MNA3	1129.11	MNA1, MNA2
CIRS Tot.	MNA1	1041.06	MNA2, MNA3
	MNA2	1472.25	MNA1, MNA3
	MNA3	1729.74	MNA1, MNA2
CIRS ICC	MNA1	1217.73	MNA2, MNA3
	MNA2	1674.86	MNA1, MNA3
	MNA3	1890.59	MNA1, MNA2
CIRS ISC	MNA1	1208.58	MNA2, MNA3
	MNA2	1669.98	MNA1, MNA3
	MNA3	1914.41	MNA1, MNA2

MMSE, Mini-Mental State Examination; GDS, Geriatric Depression Scale; ADL, Barthel Index of Independence in Activities of Daily Living; IADL, Instrumental Activities of Daily Living; MNA, Mini Nutritional Assessment; POMA, Performance-Oriented Mobility Assessment; CIRS Tot., Cumulative illness Rating Scale Total score; CIRS ICC, Cumulative Illness Rating Scale Comorbidity Index; CIRS ISC, Cumulative Illness Rating Scale Severity Index; MNA1, MNA score ≥ 24; MNA2, MNA score 23.5–17; MNA3, MNA score < 17.

A stepwise multiple regression, conducted with the least squares method, considered GDS, ADL, IADL, POMA (*p* < 0.0001), and CIRS Tot. (*p* = 0.0332) as regressors of MNA; the other variables were not included in the model (Table 6).

**TABLE 6:** Multiple regression stepwise.

	MNA		
Variables *	Coefficient	*t*	*P*
GDS	−0.29	−15.14	<0.0001
ADL	0.04	7.89	<0.0001
IADL	0.37	9.05	<0.0001
POMA	0.06	4.07	<0.0001
CIRS Tot.	−0.03	−2.13	0.0332

*Age, gender, MMSE, CIRS ICC, CIRS ISC were not included in the model (*p* > 0.1). MMSE, Mini-Mental State Examination; GDS, Geriatric Depression Scale; ADL, Barthel Index of Independence in Activities of Daily Living; IADL, Instrumental Activities of Daily Living; MNA, Mini Nutritional Assessment; POMA, Performance-Oriented Mobility Assessment; CIRS Tot., Cumulative Illness Rating Scale Total Score; CIRS ICC, Cumulative Illness Rating Scale Comorbidity Index; CIRS ISC, Cumulative Illness Rating Scale Severity Index.

For the purpose of further examining the role of the nutritional status in influencing the other CGA domains, Table 7 shows that malnutrition revealed a significant association (*p* < 0.0001) with cognitive impairment (OR: 2.09, 95%CI 1.76–2.5), depression (OR: 4.97, 95%CI 4.05–6.09), dependence on ADL (OR: 19.8, 95%CI 10.8–36.1) and IADL (OR: 7.04, 95%CI 5.58–8.89), and risk of fall (OR: 5.16, 95%CI 4.29–6.22).

**TABLE 7:** Odds ratios MNA.

Variables		MNA		OR	95% C.I.	*z*	*P*
		<17	≥17				
MMSE	≥**24**	838	316	2.09	1.76 – 2.50	8.27	<0.0001
	<**24**	1782	320				
GDS	≤**5**	407	306	4.97	4.05 – 6.09	15.43	<0.0001
	>**5**	1805	285				
ADL	≥**75**	900	522	8.75	7.03 – 10.88	19.495	<0.0001
	<**75**	1720	114				
	≥**50**	1943	625	19.8	10.80 – 36.10	9.71	<0.0001
	<**50**	677	11				
IADL	≥**6**	158	198	7.04	5.58 – 8.89	16.46	<0.0001
	<**6**	2462	438				
POMA	≥**20**	448	328	5.16	4.29 – 6.22	17.315	<0.0001
	<**20**	2172	308				

MNA, Mini Nutritional Assessment; OR, Odds Ratio; C.I., Confidence Interval; MMSE, Mini-Mental State Examination; GDS, Geriatric Depression Scale; ADL, Barthel Index of Independence in Activities of Daily Living; IADL, Instrumental Activities of Daily Living; POMA, performance-Oriented mobility Assessment.

## Discussion

We collected and examined data from a sample of 3,260 subjects referred from January 2009 to December 2020. The primary aim of this study was to examine the performance status presented by geriatric patients who turn to outpatient service, based on the components of the CGA. Understanding this aspect is fundamental to be able to build appropriate assistance and care plans. In our population, only 15.5% of the patients showed intact cognitive function, and 25.4% showed adequate mood; the percentages of the subjects that considered independent in performing basic and IADL were 15.2 and 10.9%, respectively. Low or absent increased risk of fall was found in 23.9% of the patients, and adequate nutritional status was found in 19.5%. These data denounce that too often, the people come to the attention of the specialist clinician when their status is compromised, leaving less room for maneuver to interventions, whether pharmacological or not. To confirm this thesis, it can be seen that only 2.24% of the study population could be considered “fit” in one of the CGA areas, such as cognitive, affective, functional, and nutritional areas; moreover, unfortunately, only 27.2% of functional-dependent people showed adequate cognitive performances.

About the gender-related differences in CGA scores, statistically significant in GDS, ADL, CIRS Tot., MNA, and POMA, are actually not clinically significant, except for GDS. By way of illustration, the median of ADL scores was 73 in men and 70 in women: this difference, although showing *p* = 0.0003, does not evidence any variation from the point of view of real dependence in performing daily living activities. On the other way, as mentioned earlier, GDS scores were higher, highlighting a more deflected mood, in women (median: 9 vs. 7), and this difference cannot fail to be considered relevant also from the clinical point of view, as well as pharmacological. Nevertheless, this data are consistent with the literature (29–31).

Regarding the correlations between the variables, as expected (28), CIRS Tot., CIRS ICC, and CIRS ISC were mutually very strongly correlated with each other—since a single index is insufficient (32), we also summarized the principal comorbidities—, and also the strong relationship between ADL, IADL, and POMA is consistent with the literature (14, 33). It is interesting to underline that MNA is the most correlated variable with ϱ > 0.4: it is correlated with GDS, ADL, IADL, and POMA.

Given this correlation, but also the compromised state of the population, and the limitation of the areas of intervention, the secondary aim of this study was to observe the association between nutritional status and the other CGA areas: we have chosen to look more deeply at this domain—assessed with MNA—due to its offering possibilities for intervention, according to the literature (14, 34, 35). Another domain with different possibilities of intervention could be the functional one, but these are limited, in our study population, by the cognitive impairment showed by the patients, as mentioned earlier (7, 36).

We performed an analysis to study the difference in mean ranks of the various CGA tests in three groups, namely, MNA1 (well-nourished people), MNA2 (people at risk of malnutrition), and MNA3 (malnourished people). Every variable showed the same pattern that is a worsening of the performed results to the worsening of the nutritional status. To better explain this trend, the multiple regression highlighted GDS, ADL, IADL, POMA, and CIRS Tot. as significant regressors of MNA. The strength of the association between the malnutrition and the various outcomes suggested by CGA was measured through ORs. The most powerful associations emerged with ADL, IADL, and POMA: it is intuitive, as well as confirmed in clinical studies (37–39), that malnutrition, linked to physical weakness and sarcopenia, affects functional ability, which in turn increases fall risk. It is interesting to also highlight a clear association between malnutrition and depressed mood, consistent with other previous studies (39–41). The lowest OR (2.09, *p* < 0.0001) emerged between malnutrition and cognitive impairment: although it is confirmed that a poor nutritional status impacts cognitive function, MMSE was excluded by stepwise multiple regression (variable removed by the model if *p* > 0.1) and also showed poor correlation with MNA (ϱ = 0.255, *p* < 0.0001), our data have not been able to clearly comply with this evidence (19).

This real-life, cross-sectional study analyzed the performances of geriatric people evaluated in an outpatient setting. We have sadly become aware of the delay for patients to undergo a holistic geriatric assessment for the first time: this delay can strongly limit every area of intervention.

One of the fields more correlated to the others, and in which it is easier to intervene, is the nutritional one, which is also strongly associated with the other CGA domains: our data seem to suggest that maintaining an adequate nutritional status is strongly associated with a maintenance of affective and functional status, and also of the general health of the elderly people.

The strengths of the study are represented by the size of the sample, as well as its design: the real-life approach allows to have an effective idea of the conditions of multimorbidity and demand for care of the elderly population. The main limitation of the study is represented by the fact it is single-center and did not prospectively examine the patients: in this sense, by longitudinally monitoring the patients, it would also be possible to establish causal associations between the variables.

## Data availability statement

The raw data supporting the conclusions of this article will be made available by the authors, upon reasonable request.

## Ethics statement

The studies involving human participants were reviewed and approved by the Ethics Committee of the University of Cagliari (certification NP/2022/1382). The patients/participants provided their written informed consent to participate in this study.

## Author contributions

FS, SL, and AM were principal investigators and contributed to the study design, data analyses, interpretation of the findings, and wrote the manuscript. FZ and LS contributed to the data collection. MP carried out the data analyses. All authors have read and approved the final version of the manuscript.

## Abbreviations

CGA: Comprehensive Geriatric Assessment
BMI: Body Mass Index
MMSE: Mini-Mental State Examination
GDS: Geriatric Depression Scale
ADL: Barthel Index of Independence in Activities of Daily Living
IADL: Instrumental Activities of Daily Living
MNA: Mini Nutritional Assessment
MNA1: MNA score ≥ 24
MNA2: MNA score 23.5–17
MNA3: MNA score < 17
POMA: Performance-Oriented Mobility Assessment
CIRS Tot.: Cumulative Illness Rating Scale Total score
CIRS ICC: Cumulative Illness Rating Scale Comorbidity Index
CIRS ISC: Cumulative Illness Rating Scale Severity Index
IQR: Interquartile Range
OR: Odds Ratio.
